# Applying analytic hierarchy process to assess healthcare-oriented cloud computing service systems

**DOI:** 10.1186/s40064-016-2686-3

**Published:** 2016-07-08

**Authors:** Wen-Hwa Liao, Wan-Li Qiu

**Affiliations:** Department of Information Management, Tatung University, Taipei, Taiwan; MacKay Memorial Hospital, Taipei, Taiwan

**Keywords:** Analytic hierarchy process (AHP), Cloud computing, Health care service systems

## Abstract

Numerous differences exist between the healthcare industry and other industries. Difficulties in the business operation of the healthcare industry have continually increased because of the volatility and importance of health care, changes to and requirements of health insurance policies, and the statuses of healthcare providers, which are typically considered not-for-profit organizations. Moreover, because of the financial risks associated with constant changes in healthcare payment methods and constantly evolving information technology, healthcare organizations must continually adjust their business operation objectives; therefore, cloud computing presents both a challenge and an opportunity. As a response to aging populations and the prevalence of the Internet in fast-paced contemporary societies, cloud computing can be used to facilitate the task of balancing the quality and costs of health care. To evaluate cloud computing service systems for use in health care, providing decision makers with a comprehensive assessment method for prioritizing decision-making factors is highly beneficial. Hence, this study applied the analytic hierarchy process, compared items related to cloud computing and health care, executed a questionnaire survey, and then classified the critical factors influencing healthcare cloud computing service systems on the basis of statistical analyses of the questionnaire results. The results indicate that the primary factor affecting the design or implementation of optimal cloud computing healthcare service systems is cost effectiveness, with the secondary factors being practical considerations such as software design and system architecture.

## Background

The US National Institute of Standards and Technology (NIST) defines (Mell and Grance 2011) cloud computing as “a model that enables ubiquitous, convenient, and on-demand network access to a shared pool of configurable computing resources (e.g., networks, servers, storage, applications, and services) that can be rapidly provisioned and released with minimal management effort or service provider interaction.” Cloud computing technology has been increasingly applied in healthcare settings in recent years. Because of the rapid advancement of information and communication technologies, which are gradually being applied to healthcare systems, so-called healthcare cloud platforms integrating networking technology applications are expected to increase in prevalence, eventually replacing existing healthcare operating models.

Cloud computing services remain at conceptualization stages at most Taiwanese medical institutions. In addition, shifting from traditional to cloud-computing-based service models poses a challenge for healthcare organizations. Hence, developing a systematic strategic evaluation model for medical organizations would facilitate the design of cloud computing systems and the ranking and prioritization of management indicators; this can assist administrators in decision making and consequently enhance the efficiency of assessing cloud computing service systems, ensure the effective allocation of limited resources, and prevent unnecessary resource replacement and waste. Therefore, the current study adopted the analytic hierarchy process (AHP), a technique that is widely used in the decision and management fields (Saaty 1980). This study conducted a literature review and expert questionnaire survey to identify critical factors affecting the construction of cloud computing services in the healthcare industry. Specifically, the study procedures involved developing a hierarchical structure, designing an AHP questionnaire, and calculating the weights of hierarchy variables on the basis of the results of the expert survey. Through the literature review and expert survey, this study investigated cloud computing technology, identifying the crucial factors influencing the assessment of cloud computing service systems for medical institutions, and determined how experts and administrators ranked and prioritized management indicators to facilitate decision making.

## Literature review

### Cloud computing

“The cloud” comprises parallel and distributed systems in the dynamic deployment of interconnected virtual computers. Service providers establish service-level agreements (SLAs) on the basis of unified computing resources to achieve a consensus with consumers (Buyya et al. 2008). The cloud is also a metaphorical resource pool that facilitates consumer access to virtual resources (e.g., hardware, development platforms, and services), and such resources can be dynamically reconfigured and adjusted in size according to their load conditions to optimize resource utilization. Regarding the resource pool, service providers achieve a consensus with consumers on the basis of SLAs, providing guarantees according to consumer demands and required payments (Vaquero et al. 2008).

According to the NIST definition, cloud computing can be categorized into three types on the basis of the service model for resource provision: infrastructure as a service (IaaS), platform as a service (PaaS), and software as a service (SaaS) (Kuo 2011).

SaaS is a service model in which consumers access services supplied by service providers (e.g., customer relationship management and enterprise resource planning systems) by using a web browser or other systems through an Internet connection. Consumers are not required to consider system performance levels or storage, but they are responsible for software license fees and must make adjustments according to the software usage methods. Moreover, consumers are not required to adopt the traditional software licensing model and are exempt from risks of implementation failure. If software installation problems or ineffective usage persists, enterprises can terminate services to reduce sunk costs.

PaaS is a service model that provides a platform interface for information technology (IT) developers. In this model, consumers can employ upload or application programming interfaces supplied by service providers to upload their self-written programming codes for online usage. Examples of PaaS services include Amazon EC and Amazon S3. The PaaS model provides consumers with platform and deployment configuration services, relieving them of the burden of routine system maintenance or complex deployment issues.

IaaS is a service model that offers a service platform for information infrastructure deployment. In this model, consumers purchase virtual infrastructure services according to their needs (e.g., host computers and firewalls). Moreover, this service model satisfies short-term and temporary demands. Service providers establish virtual resource pools, providing consumers with dynamically adjustable resources as well as pay-per-use and unlimited client services. Consumers can access enterprise-level IT infrastructures and resources, saving them the expenses and constant capital expenditures required to implement such infrastructures.

Regarding cloud computing adoption decisions, a previous study surveyed enterprises, and the survey results revealed that 71 % of the enterprises reported that cloud computing is a technological choice, 70 % reported that it enabled flexible business operations, and 62 % reported that it facilitated rapid responses to market conditions in addition to reducing implementation expenses by 65 %, thereby enhancing their core business operations (Misra and Mondal 2011).

Because the objectives of medical institutions differ from those of for-profit businesses, the benefits and effects of cloud computing systems cannot be assessed on the basis of the perspectives of for-profit businesses. Nevertheless, limited healthcare resources render government resource subsidies alone inadequate to satisfy hospital management requirements. Therefore, medical institutions should not be confined by conventional notions, and they should consider a perspective of increasing profits and competitive advantage to develop service models that are aligned with contemporary trends and assist competition among international and domestic peers. In this context, service models for healthcare-based cloud computing are the optimal choice for development.

### Relationship between Medical Institutions and cloud computing systems

Health information systems comprise computers and communication devices, which collect, store, process, and retrieve management information related to patient care and hospital activities, satisfying the requirements of authorized users regarding hospital information management systems (Shortliffe et al. 2001; Tsai and Yang 2006). Based on the definition of hospital information systems, a complete system should involve the following functions: (a) basic hospital operations; (b) business and finance; (c) communication and networks; (d) division and department management, including providing the information required by individual departments; (e) medical documentation, including collecting standard medical records, in addition to organizing, storing, and displaying clinical information for individual patient care; and (f) medical decision support, involving the collection of various data to facilitate patient diagnosis and treatment.

The healthcare industry is highly specialized and knowledge-intensive, and patient diagnoses and examinations often yield substantial amounts of data. Medical personnel make judgments on the basis of the examination results before providing care, such as medication adjustments, to achieve effective patient treatment. To accommodate increasing amounts of examination data and application requirements, numerous organizations have explored how to apply cloud computing models to health care (Kim 2009; Motahari-Nezhad et al. 2009).

The major difference between traditional IT systems and cloud computing systems are elucidated subsequently. In traditional IT service models, the relationship between business applications and physical computing resources is a one-to-one, static mapping relationship. Each application system comprises fixed physical IT resources (e.g., servers, operating systems, and storage space) that operate the overall system. By contrast, in cloud computing IT service models, the relationship between business applications and physical computing resources is a dynamic mapping relationship, in which resources are delivered as required. Consumers are not required to know which physical server corresponds to the operations of an application system. In addition, sharing resources and services can substantially reduce IT investment costs, enhance overall usage efficiency, and strengthen system reliability, thus improving the service standards of IT departments and increasing the service flexibility of medical institutions. Cloud computing technology is expected to grow in prevalence; moreover, private clouds for pharmacies, clinics, medical centers, regional and district hospitals, and health insurance bureaus can be interconnected to create an integrated healthcare-oriented cloud computing network.

Telehealthcare is one of the cloud-computing-based services proposed by the Medical Cloud Project organized by the Department of Health under the Executive Yuan (presently called Ministry of Health and Welfare, Taiwan); other cloud-based services include personal healthcare information provision, medical record backup, and public hospital healthcare cloud services. Studies conducted by the Council for Economic Planning and Development indicated that the population aged older than 65 constituted more than 10 % of the total population of Taiwan in 2008, and this number is projected to reach 14 % in 2020; moreover, in 2015, the total output value of the worldwide healthcare industry was projected to reach US$597 billion (Taiwan’s National Health Insurance 2016).

For strategic planning and policy implementation, applying a strengths, weaknesses, opportunities, and threats (SWOT) analysis to assess healthcare-oriented cloud computing service systems cannot provide an effective direction or facilitate the establishment of a two-way error correction mechanism for the current development and future planning of healthcare cloud computing services (Kuo 2011).

The findings of studies conducted using the AHP can assist policy makers in understanding the priorities of all decision alternatives. Alternatives with higher weights are assigned higher priorities, reducing the risks associated with wrong policy decisions (Spires 1991; Saaty 1980). The AHP is suitable for application in the following processes: (1) planning, (2) generating decision alternatives, (3) determining priorities, (4) choosing optimal plans or policies, (5) determining resource distributions, (6) identifying needs, (7) executing outcome prediction or risk assessment, (8) implementing system designs, (9) executing performance evaluations, (10) stabilizing systems, (11) achieving optimization, (12) and engaging in conflict resolution (Saaty 1980).

The AHP is a systematic framework that simultaneously establishes a hierarchical structure exhibiting interacting levels, compares the importance of various factors, and assesses decision alternatives under the existing structure; the most prominent advantage of the AHP is its ability to structuralize, hierarchize, and quantify decision problems as a systematic assessment model, providing a reference for policy makers addressing multicriteria decisions or standards (Saaty 1980, 1990, 1999). In general, the AHP follows the following basic principles:

A system can be divided into several classes or components and can be developed into a network-like hierarchical structure. In a hierarchical structure, the elements on each level are assumed to be independent.

1. The elements on each level can be evaluated on the basis of certain or all elements (which can function as a criterion) from the level above.
2. During the comparison or assessment of elements, measurements on a standard absolute value scale can be transformed into measurements on a relative ratio scale.
3. Positive reciprocal matrices can be used for pairwise comparisons of the elements on each level.
4. Preference relations satisfy transitivity, and transitivity can be explained by the following relationship: If A exhibits greater importance than B and B demonstrates greater importance than C, then A definitely exhibits more importance than C.
5. Full transitivity is not always practical; hence, intransitivity also exists. However, the consistency of intransitivity must be tested.
6. The priorities of the elements are calculated according to weighting principles.

The preceding analysis demonstrates the advantages and importance of healthcare cloud computing service models. To plan cloud computing service systems for health care, numerous influential factors must be considered. Developing a systematic strategic evaluation model for medical institutions would facilitate assessments relevant to cloud computing system planning and the ranking and prioritization of management indicators; this can assist administrators in decision making and consequently enhance the efficiency of cloud computing service system assessments, in addition to ensuring the effective allocation of limited resources.

Previous studies have employed the AHP method to assess the suitability of various systems for cloud computing, providing solutions based on the load application needs of the systems. The research also compared the weights of each hierarchical level allocated by an enterprise, and suggestions were provided regarding the optimal decision framework for cloud computing application processes (Brust and Sarnikar 2011).

A comprehensively designed cloud computing service system must integrate the knowledge and experience of medical and information management personnel to be effectively applied in healthcare settings. Anti-intrusion measures are vital to cloud computing systems, particularly since the enactment of the revised Personal Information Protection Act in October 2012. If personal data stored in cloud databases are leaked, the privacy and interests of patients can be compromised, and this can expose hospitals to the risk of incurring losses engendered by considerable penalties or compensation fees.

## Methods

**Fig. 1:**
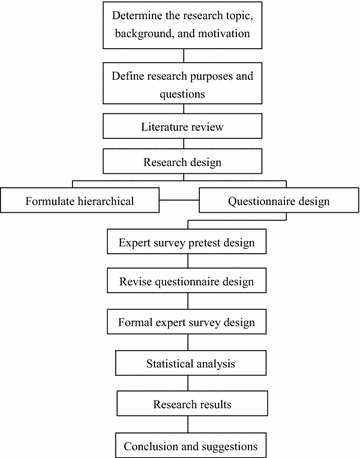
Research framework

Figure 1 presents the research framework employed in this study. The analysis results can provide a reference for healthcare professionals.

The preliminary questionnaire was revised on the basis of the pretest results, and variables irrelevant to cloud computing service systems in the healthcare industry were excluded. Table 1 presents the variables adopted in this study.

**Table 1:** Framework of hierarchy variables

Dimension	Influential variables
Health care cloud software	Convenient software, software scalability, cloud-based medical image exchange, integration of information and health care services
Cloud service delivery	Data storage security, system stability, software research and development, testing and debugging
Cloud management issues	Cost-effectiveness, flexible and expandable framework, convenient information sharing, regulatory compliance

The variables are defined as follows:

1. Convenient software: Using web-based software requires no complex input or procedures. The functions of web-based software can be quickly configured and executed.
2. Software scalability: The software design meets the requirements of a healthcare environment, enabling patients or staff members to alter its functions or configuration, in addition to satisfying changing user demands.
3. Cloud-based medical image exchange: In addition to exchanging existing electronic medical records, various hospitals can cooperate and coordinate among one another, developing cloud exchange agreements for medical images.
4. Integration of information and healthcare services: Personnel implementing information systems must communicate with healthcare staff members, leveraging their work experience and expertise to adequately examine and discuss system requirements; doing so enables implementing an appropriate information system that satisfies user needs.
5. Data storage security: Cloud databases should include information security and antivirus protection measures as well as adequate data backup systems to ensure data integrity and normal operations during power failures or other disasters.
6. System stability: Cloud computing systems require comprehensive data security and protection systems to avoid system failure engendered by viruses and cyber-attacks. Such systems should also possess sufficient capacity to accommodate considerable increases in data traffic and provide effective connection to data backup systems. The backup systems should provide timely support in the event of database failures.
7. Software research and development: Research and development of software for the web-based operation of cloud computing systems can be undertaken.
8. Testing and debugging: In addition to testing and modifying newly developed software programs, routine operational and anti-intrusion testing should be performed to detect hacking attempts or other aberrant system behaviors. Any abnormalities should be addressed and resolved immediately.
9. Cost effectiveness: This refers to the savings from purchasing maintenance and management software, data storage devices, or other hardware. This also pertains to the savings from custom-designing network systems, following the adoption of cloud computing technology.
10. Flexible and expandable framework: Healthcare cloud computing systems can be adjusted or modified on the basis of end user (healthcare personnel or patients) opinions, and new functions can be added to expand the original system architecture.
11. Convenient information sharing: When patients transfer to other clinics or hospitals, medical staff members can conveniently exchange records regarding patient physiological conditions (e.g., blood pressure and blood glucose level) and relevant test reports.
12. Regulatory compliance: Cloud computing systems should be designed or configured to comply with relevant national policies and regulations specific to health care. For example, the acquisition of personal information and cloud-based exchange of medical records should comply with the Personal Information Protection Act of Taiwan.

Previous studies have employed the AHP to explore critical factors associated with the successful implementation of cloud computing services in the healthcare industry, revealing that the first hierarchical level of critical factors comprised five dimensions, which (ranked in order of increasing weight) are as follows: organizational culture, industry planning, project management, technical service capability, and leadership. Leadership was the most critical dimension, within which effective project teamwork capability was the most vital (Chen and Chang 2012). Therefore, effective project teamwork is required to design health information systems and promote and implement cloud computing service systems. Regarding cloud computing for electronic health services, a study identified crucial assessment factors by conducting a systematic literature review (Hage et al. 2013). Searches were conducted using the EBSCO1, EBSCO2, Embase, MUSE, and Web of Science databases and specific keywords, namely *e*-*Health*, *e*-*care*, tele*, *ICT*, *information technology*, *communication technology*, *communication system*, and *information system*. Of 5896 abstracts yielded by the keywords, 51 articles met the requirements of the researchers, enabling the identification of various dimensions including rural context, implementation process, e-Health content, and adoption outcomes (Hage et al. 2013). Related research indicated that the scalability of cloud computing platforms is a key factor for the successful application of such platforms in electronic health record systems, and this is because scalability determines the service quality (Vilaplana et al. 2013). Acknowledging the trend in cloud computing systems for health care, Microsoft Corporation (Microsoft 2014), which provides comprehensive cloud computing platforms, has developed related business models of health clouds; Microsoft Corporation stated three emphases for such systems: security and privacy, reliability, and flexibility and interoperability.

Decision Evaluation Model for Healthcare-based Cloud Computing Systems:

*Healthcare cloud software* Convenient software, software scalability, cloud-based medical image exchange, and integration of information and healthcare services.

*Cloud service delivery* Data storage security, system stability, software research and development, and testing and debugging.

*Cloud management issues* Cost-effectiveness, flexible and expandable framework, convenient information sharing, and regulatory compliance

**Fig. 2:**
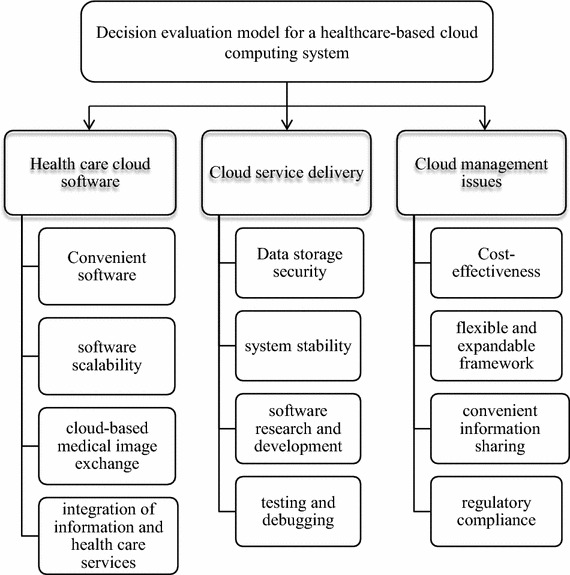
Framework of the AHP hierarchy

Figure 2 illustrates the framework of the hierarchy variables established by filtering the preceding criteria.

## Results

To conduct a survey, this study applied the following criteria to determine whether experts to be invited to participate in the survey have professional knowledge in specific areas:

1. Do they possess more knowledge on a specific topic than most people do?
2. Do they have related work experience levels or did they work in related professional organizations?

According to these criteria, this study invited experts comprising IT scholars, specialists from various research units, representatives from the cloud computing industry, and managers from the IT departments of medical institutions. To adhere to the Personal Information Protection Act and research ethics, the personal information of the experts can only be partly disclosed as follows: The managers from the IT departments of medical institutions were professors and executive officers employed in medical centers in northern Taiwan, and the representatives from the cloud computing industry were employed in the IT departments of Chunghwa Telecom and FarEasTone Telecommunications

This study adopted the AHP to explore critical factors influencing the assessment of cloud computing systems for the healthcare industry. An expert questionnaire survey was also conducted to collect quantitative data. Before the participants completed the questionnaires, each pairwise comparison question was explained to obtain accurate results; pairwise comparison matrices were then constructed on the basis of the results. The eigenvalues and eigenvectors of each pairwise comparison matrix were calculated, and the consistency of the matrices was tested. A specific AHP statistical software program (Expert Choice) was employed to conduct statistical analyses on the questionnaire data. The questionnaires were distributed to five medical center staff members and three senior cloud technology professionals in northern Taiwan. All of the eight questionnaires distributed were returned, yielding a valid return rate of 100 %. Table 2 presents the results of the statistical analyses, including the hierarchy variables and the weights of their constituent elements.

**Table 2:** Statistical summary of the hierarchy variables, assessment criteria, and their respective weight values

Hierarchy variable	Weight	Assessment criteria	Weight	Average weight	Rank
Health care cloud software	0.325	Convenient software	0.205	0.0666	10
		Software scalability	0.284	0.0937	3
		Cloud-based medical image exchange	0.248	0.0818	8
		Integration of information and health care services	0.263	0.0868	7
Cloud service delivery	0.306	Data storage security	0.291	0.0890	4
		System stability	0.286	0.0875	6
		Software research and development	0.233	0.0713	9
		Testing and debugging	0.19	0.0581	12
Cloud management issues	0.369	Flexible and expandable framework	0.286	0.1055	2
		Convenient information sharing	0.239	0.0882	5
		Cost-effectiveness	0.299	0.1103	1
		Regulatory compliance	0.176	0.0649	11

Table 2 indicates that the weights for healthcare cloud software, cloud service delivery, and cloud management issues are 0.325, 0.306, and 0.369, respectively.

### Assessment criteria for healthcare cloud software

The analysis results indicate that the experts emphasized software scalability in the design of healthcare-oriented cloud computing service systems, in which software architecture is the core of all services. Software scalability is particularly crucial because software designs must meet the requirements of healthcare environments, enabling patients and healthcare personnel to adjust the functions or configuration of systems; meeting such requirements enables the resulting healthcare-oriented cloud computing service systems to achieve high levels of user satisfaction. Integration of information and healthcare services was also a critical factor, and this is because healthcare service systems are not simply general information systems but may require functions relevant to emergent care. Therefore, health information systems must contain architectures that facilitate rapidly and smoothly transmitting medical information and distinguishing and classifying medical images or data, thereby enabling staff members to efficiently determine the urgency of events.

### Assessment criteria for cloud service delivery

Stable cloud computing systems yield reliable and high-quality services. Additionally, stability is closely associated with system security because the degree to which system designers emphasize information security is proportional to the likelihood of system stability and data storage security being achieved. Because healthcare-oriented cloud computing service systems operate on network architectures, they remain vulnerable to hackers, viruses, and personal information theft.

### Assessment criteria for cloud management issues

Cloud computing is a current trend in healthcare services and is partially motivated by cost-saving concerns. Adopting a cloud computing system can result in various savings; the more the expenses an adopted cloud computing system saves, the greater is the system’s effectiveness. For example, the SaaS model allows enterprises to pay software usage fees monthly and purchase version updates according to the number of system users, thereby reducing IT expenses. Hence, as long as hospitals pay monthly license fees, they can prolong the lifecycle of software products without being bound to traditional software licensing models or bearing the risks of product ineffectiveness. The IaaS model provides information infrastructure service platforms, allowing consumers to purchase infrastructure-related services, such as host computers and firewalls, without purchasing a physical infrastructure; this model saves consumers’ hardware expenses. In addition, service providers supply dynamically adjustable, pay-per-use, and unlimited client services by establishing virtual resource pools, saving consumers the expenses of designing, implementing, and maintaining customized infrastructures. Cloud services also eliminate the expenses associated with purchasing maintenance and management software, data storage devices, or other hardware. Without cloud services, consumers must bear the expense of maintaining and upgrading hardware and software. Therefore, cost effectiveness is a critical factor in the assessment of healthcare-oriented cloud computing service systems.

The flexibility of a cloud computing system is equivalent to its expandability; the system can be adjusted according to the opinions of end users (healthcare personnel or patients), or new functions can be added to it. Consequently, the system effectively satisfies market needs and gains user acceptance, creating brand value for the hospital.

### Summary of assessment results

Regarding the average weights of the variables, cloud management issues demonstrated a relatively high value. The other criteria that surpassed the 50th percentile were software scalability, data storage security, convenient information sharing, and system stability. Cost effectiveness, which was related to financial considerations, proved the most influential factor for designing or configuring healthcare-oriented cloud computing service systems. A flexible and expandable framework and software scalability, which was related to client services, ranked second to cost-effectiveness.

## Conclusion

The results of this study show that cost effectiveness is a critical factor in the assessment of healthcare-oriented cloud computing service systems; practical considerations related to software design and system architecture are of secondary importance.

### Management implications

When planning information systems, enterprises evaluate return on investments (ROIs) to analyze the benefits of system implementation, calculating investment expenditure to determine whether an implementation project is worthwhile. A positive ROI indicates that the proposed system implementation is worthwhile, whereas a negative ROI indicates that the investment cannot achieve the desired outcome. Discounting specific installations that are necessary, enterprises typically avoid implementing systems that cannot justify the investment cost. Although medical institutions are not-for-profit organizations, they should consider decision-making processes similar to those of for-profit businesses regarding the planning and assessment of healthcare-oriented cloud computing service systems. Decision makers should carefully consider the amount of cost savings that adopting cloud computing services can yield. Such savings are associated with the elimination of expenses concerning the acquisition of maintenance and management software, data storage devices, and other hardware; independent implementations of network system architectures; as well as software and hardware maintenance and upgrade. The opportunity costs potentially saved by adopting healthcare-oriented cloud computing service systems can be included as profits when assessing ROIs.

Next, the practical use of healthcare-oriented cloud computing systems is considered. According to the statistical results, software scalability is essential because software designs must enable end users to adjust the functions or configurations of software systems, in addition to satisfying user requirements; doing so can yield high levels of user satisfaction. In addition, healthcare-oriented service systems are not typical information systems; healthcare and information management staff members should thus thoroughly investigate and discuss various issues, including how systems can rapidly and smoothly transmit health information, and distinguish or classify medical images and data to facilitate the prioritization of clinical procedures. Consequently, information and healthcare services can be integrated by consolidating the knowledge and experience of healthcare and information management personnel, thereby creating comprehensive healthcare-oriented cloud computing service systems.

Finally, from a service perspective, the current healthcare market is consumer oriented. The healthcare-oriented cloud computing industry is part of the service industry. Hence, whether cloud computing systems are user-friendly, satisfy user requirements, and possess expandability or flexibility is vital to their success. Such systems should be adjustable or expandable on the basis of the opinions of end users (healthcare staff members or patients). Consequently, the resulting systems can accommodate market demands and obtain user acceptance, generating brand value for hospitals.
